# Adolescent Internet gaming addiction and personality characteristics by game genre

**DOI:** 10.1371/journal.pone.0263645

**Published:** 2022-02-10

**Authors:** Dongil Kim, JeeEun Karin Nam, Changmin Keum

**Affiliations:** 1 Department of Education, Seoul National University, Seoul, South Korea; 2 Graduate School of Education, Ewha Womans University, Seoul, South Korea; 3 Department of Counseling & Psychotherapy, Inje University, Gimhae, South Korea; University of Hradec Kralove: Univerzita Hradec Kralove, CZECH REPUBLIC

## Abstract

With the emergence of a new concept called ‘Internet Gaming Disorder’ in DSM-5 and ICD-11, related research is underway around the globe. However, not enough literature on Internet gaming addiction has considered the diversity in game genres. Internet gaming addiction may take on a different form according to the particular characteristics of a game being played. To better understand adolescent Internet gaming addiction, this study sought to identify the differences in Internet gaming addiction and personality characteristics based on the game genre played. A total of 3,217 elementary and middle school students across Korea participated in the survey that included the Maladaptive Game Use Scale and the Adolescent Personality Questionnaire. ANOVA analysis revealed that Internet gaming addiction and personality characteristics varied according to the game genre played. In particular, post-hoc tests showed that Real-Time Strategy (RTS) and First-Person Shooter (FPS) game users have higher levels of tolerance, withdrawal, and neglect of everyday life compared to other genres such as Role-Playing Game (RPG), Racing, and Arcade/Shooting. Also, Internet gamers users of particular genres showed significantly lower self-esteem (Arcade/Shooting), conscientiousness (Racing), empathy (RPG), and sense of community (Racing and RTS) scores than comparison group. The implications of the study results were discussed with a special emphasis on Internet gaming addiction interventions.

## Introduction

With the development of the Internet and electronic devices, Internet gaming has become a familiar leisure activity. Appropriate Internet gaming can have a positive impact on leisure time, friendship, problem-solving skills, short-term memory enhancement [1–3], but excessive gaming to the extent that users lose self-control can interfere with their studies [4–6], increase impulsiveness and aggression [7–9] and cause alienation in real-life relationships [10, 11]. The DSM-5 published by the American Psychiatric Association [12] proposes diagnostic criteria for Internet Gaming Disorder as preoccupation with gaming, withdrawal, tolerance, failure to control playing, loss of interest in other activities, continued excessive use despite its consequences, and lying about the amount of gaming time, etc. This indicates that the symptoms and mechanisms of Internet gaming addiction may be similar to substance-related addictions [13, 14], the most prominent of which is that game users feel that they cannot control craving for games and gaming behavior [15]. Also, addicted gamers wish to play more and more, and when they do not play, they experience withdrawal symptoms that create feelings of discomfort. Moreover, the World Health Organization [16] has classified gaming disorder as a new disorder in its recently revised ICD-11. The loss of control over gaming behavior and the game’s priority over other interests and daily activities, and the continuation of game playing despite its negative impacts were seen as symptoms. While Internet gaming addiction research still needs to be significantly scaled up [12, 17], reports of the side effects of Internet gaming are accumulating warrant for detailed and effective countermeasures.

With the rise of Internet games, the game industry has been ever expanding. Subdivided and diversified, game genres range from strategic simulation games, in which gamers collaborate to develop strategies, to arcade games, in which a single gamer performs simple tasks. As such, several studies have sought to understand how game characteristics as well as the gamers’ motivations and other psychological variables differ according to game genre. Based on prior studies that suggested that the structural characteristics of gambling act as a psychological mechanism for addiction, King et al. [18] classified the structural characteristics of games into social features, manipulation and control features, narrative and identity features, and reward and presentation features. Further, they suggested that differences in these structural characteristics among games may contribute to the variations in the psychological mechanism of excessive gaming [18, 19]. Griffiths [20] also asserted that gaming addiction is not caused by the smart media itself or device factors, but rather by the characteristics of each game. Hilgard, Engelhardt, & Bartholow [21] found that video gamers’ motivation and preferences can be classified into Story, Violent Catharsis, Violent Reward, Social Interaction, Escapism, Loss-Sensitivity, Customization, Grinding, and Autonomy, and looked for factors that lead to their preference for particular games.

For example, in the case of Real-Time Strategy (RTS) games (e.g., "StarCraft," "Company of Heroes"), two or more players engage in real-time brain battles according to set rules, requiring quick and accurate judgment, analytical skills and meticulous strategic thinking to achieve goals [22]. For these RTS games, studies show that individuals with higher need for recognition in virtual reality, greater aggressiveness, and lower desire for interpersonal relationships are more likely to become addicted to gaming [23]. In the case of Massive Multiplayer Online Role-Playing Game (MMORPG) games (e.g., “World of Warcraft,” “Final Fantasy XIV”) multiple players each form a unique avatar and simultaneously interact with each other to achieve a given task (quest) in the online environment [24, 25]. For these MMORPG games, interaction with other users, problem solving skills, collaboration and leadership are required [26–28] and individuals with greater motivation to release aggression and to escape the reality are more likely to be addicted [29, 30]. First-Person Shooter (FPS) genre (e.g., "Overwatch," “Battle Ground") includes games where a player literally uses his or her weapon from a first-person point of view to overcome the opponent. Because the game player and the game avatar share the same perspective, FPS games are easy to manipulate and feel real and intense, but they require players to be aware of reciprocity based on mutual cooperation among players [31]. The violent scenes and sounds of FPS games that feel highly realistic can stimulate emotions and thoughts associated with the player’s aggression [32], and virtual world imitations that flow into the real world may pose serious problems [33]. Other genres include sports, racing, and arcade, which are game genres that borrow the form of early video games. These are the kind of games in which one-off victory or defeat is determined by a one-on-one match, puzzle, or board game. There is no need to develop complex strategies, but it is important to focus and win in a short time using the eye-hand coordination ability [34]. As such, games have different characteristics depending on the genre. As game characteristics interact with the player characteristics in affecting gaming behaviors, Internet gaming disorder may be more likely for people with certain vulnerabilities.

Studies have also shown that differences in addiction symptoms exist according to game genre [35–39]. For instance, MMORPG and FPS games appear to have a stronger correlation with Internet gaming addiction than other genres [40–43]. At the same time, studies have noted the differences in psychological and social characteristics that exist among game users depending on the game genre mainly played. One study compared users of FPS and strategy games and discovered that FPS experience was linked positively with impulsivity, while strategy game experience was correlated negatively with impulsivity [44]. In another study, FPS players showed significantly higher levels of internet gaming addition than users of action and simulation games, as well as lower levels of self-control than users of other genres [39]. Such differences in individual game user characteristics depending on the game genre preference may contribute to the differences in addiction symptoms.

Some studies have attempted to reveal the relationship between game genres and individual characteristics, but not enough research has been done to keep up with the increasing diversification of games due to the rapid growth of game industry [45]. Montag et al. [46] discovered that German male youths who mostly played FPS games had low self-directedness. Graham and Gosling [47] examined studied the personality traits of MMORPG players based on their motivation for playing. Those who played MMORPGs for socialization were more extroverted, agreeable, neurotic, and open to new experiences, whereas those who played MMORPGs for achievement were more extroverted, neurotic, and open to new experiences, but less agreeable and conscientious. Lee et al. [48] examined the interpersonal patterns of Korean teenagers according to the game genre, and found that simulation, RPG, and shooting game users prefer to play in groups more than board game or sport game users. Braun et al. [45] reported that people who prefer action game genres exhibit significantly lower levels of neuroticism and higher levels of extroversion than those who prefer role-playing or simulation games. If indeed significant differences exist in symptomatic and personality characteristics among gamers by the game genre they mostly play, then a more effective intervention will be one that takes these differences into consideration. Therefore, this large-scale exploratory study sought to comprehensively examine the relationship between Internet gaming addiction symptoms and personality characteristics of adolescents by game genre.

## Methods

### Sampling procedure and participants

According to the Juvenile Protection Act (Article 26), a bi-annual survey on Internet game use is conducted on Korean adolescents. The data analyzed in this study was from the 2017 survey. Data collection was ethically conducted by the National Youth Policy Institute of Korea through a professional research company. Cross-national surveys conducted by government agencies such as National Youth Policy Institute are officially IRB-exempt. Internal ethical considerations were properly followed. For instance, the students of schools that have consented to participate voluntarily completed the survey after reading a written information and consent form. Students in grades 4, 5, 6, 7, 8, and 9 from seven regions (Seoul, Gyeonggi, Incheon, Busan, Daegu, Daejeon, Gwangju) of Korea were recruited. Stratified sampling method was used to better ensure the representativeness of Korean students in these grade levels, and to reduce any potential error produced by an imbalance in age or gender distribution in the sample. Participants completed paper surveys at their schools in January and February of 2017.

Responses from 3,217 students (age *M =* 11.54; *SD* = 1.70) were used in the analyses. The average game usage time was 42.03 minutes (SD = 63.65) on weekdays and 74.10 minutes (SD = 109.19) on weekends. The maximum duration of game usage was 75.25 minutes (SD = 117.61) on weekdays, and 132.74 minutes (SD = 196.63) on weekends. A detailed descriptive statistics of participants in this study are shown in Table 1 below.

**Table 1:** Descriptive statistics of study participants.

		Elementary School			Middle School			Total
		4th	5th	6th	7th	8th	9th	
Gender	Male	260	248	265	304	271	288	1636
	Female	259	263	248	258	286	267	1581
Total(by grade)		519	511	513	562	557	555	3217
Total(by school)		1543			1674			

### Measures

#### Game genres

As for the game genre most played, each participant was asked to write their top three mostly played game. The games were then classified into genres. Currently, several studies exist on game genre classification [49–52] but there is still no consensus on the matter. Indeed, genre studies do not capture the complexity of fast-developing modern games [53]. With this limitation in mind, the researchers first categorized the games based on the online game genre classification presented in the Korean Game White Paper [54]. With the rapid growth of Internet gaming industry in South Korea, there was a surge in the nationwide interest in the side-effects of gaming. In this context, the Korean government has been publishing white papers related to game usage annually. As a result, we elected to use the classification criteria proposed by the comprehensive national report on games in our research. Then. we had two game experts review the classification. This process resulted in classifying the games into six genres (RPG, FPS, RTS, Racing, Sports, Arcade/Shooting). Brief descriptions and the corresponding games of each genre are shown in Table 2 below.

**Table 2:** Game genre classification.

Game genre	Examples	Description
**RPG (Role Playing Game)**	Mapel Story, Roblox	Role-playing game. Each player takes on a character and accomplishes the role given within the game and completes various missions. Player’s character’s level is important.
**FPS (First Person Shooter)**	Overwatch, Sudden Attack,Crazy Shooting Bubble Fighter, Counter Strike, Team Fortress2	1st person shooting game. The game user becomes a character within the game and engages in battles by using various weapons to attack or defend. The game is played from the first person view, so the player’s sense of immersion is maximized.
**RTS (Real-Time Strategy)**	League of Legends, StarCraft1&2, Herosof the Storm, Clash Royale	Real-time strategy simulation game. The game user becomes a commander and leads the battle using various strategies and tactics. Keen analytical ability, strategic decisions and problem solving skills are more important than physical skills.
**Racing**	Talesrunner, Kart Rider	Racing game uses cars, motorcycles, etc. and the game user needs eye-hand coordination ability to win the race.
**Sports**	FIFA Online2, FIFA	The game user either participates in sports or strategically controls a team of players to win.
**Arcade/Shooting**	Minecraft, Elsword, Crazy Arcade	Similar to the games played in offline arcades, these games are relatively simpler than other game genres and can get game results quickly by being immersed for a short span of time.

#### Internet gaming addiction

The Comprehensive Scale for Assessing Game Behavior (CSG) developed and validated by the Korean Creative Content Agency [55] was used to measure the level of game addiction in adolescents. Although the original scale is comprised of the Adaptive Game Use Scale (AGUS) and the Maladaptive Game Use Scale (MGUS), only MGUS was used for the purpose of the study. MGUS focuses on the negative consequences of game behaviors and include 7 subfactors (Tolerance, Withdrawal, Excessive Time Spent, Regulation Damage, Compulsive Use, Neglect of Everyday Life, Continued Use despite Side Effects). Tolerance measures the extent one requires increasingly more time to play games to feel satisfied. Withdrawal measures the extent one experiences sadness, anxiety, irritability when not playing games. Excessive Time Spent measures the extent one spends undue time playing games. Regulation Damage measures the extent one fails at self-regulation in relation to game usage. Compulsive Use measures the extent one is unable to get out of thinking about gaming or game itself. Neglect of Everyday Life measures the extent one disengages from important social, academic, or leisure activities due to gaming. Continued Use despite Side Effects measures the extent one continues to play games despite various side effects. MGUS has a total of 21 items (3 items for each factor). Items were rated on a 4-point Likert scale, where higher score indicate problematic game use. In this study, the Cronbachs’ α for the overall scale was .960. As for the subscales, the Cronbachs’ alphas were .812 for Tolerance, .868 for Withdrawal, .878 for Excessive Time Spent, .909 for Regulation Damage, .731 for Compulsive Use, .736 for Neglect of Everyday Life, and .777 for Continued Use despite Side Effects.

#### Personality characteristics

Adolescent Personality Questionnaire (APQ) developed and validated by the Korean Ministry of Education [56] was used to assess students’ personality characteristics. In this scale, the adolescent personality is divided into intrapersonal and interpersonal dimensions. The subfactors of intrapersonal dimension are Self-Esteem, Conscientiousness, and Openness. The subfactors of interpersonal dimension are Empathy, Social Leadership, and Sense of Community. In total, there are 6 subfactors and 24 items, rated on a 4-point Likert scale. The overall reliability for the scale turned out to be .918 in this study. The Cronbachs’ α for the subscales were .806 for Self-Esteem, .717 for Conscientiousness, .738 for Openness, .758 for Empathy, .715 for Social Leadership, and .674 for Sense of Community.

### Data analysis

The data collected for the study was analyzed using SPSS 18.0. Descriptive statistics were calculated to show the general tendencies of game addiction and personality characteristics. Also, internal consistency reliability (Cronbach’s α) tests were run for each measure, and Pearson’s product moment correlation analysis was conducted to see the relationships among the subfactors. Finally, ANOVA and post-hoc analyses were run to see the differences in addiction and personality characteristics by game genre.

## Results

### User statistics by game & genre classification

As for the game genre most played, total of 987 different games were mentioned by the participating adolescents. Regardless of the ranking, 20 games with high frequency of appearance were derived through simple summing, and were ranked according to the frequency of responses. Among them, "Junior Naver," which ranked 18th, was exclude because it is a comprehensive game platform that provides access to games of many different genres as well as other various contents such as videos and storybooks. The final 19 games are presented in Table 3 below.

**Table 3:** Top PC online games played by adolescents.

Rank	Game Name	Genre	Elementary		Middle		Total	
			N	%	N	%	N	%
1	Over watch	FPS	105	13.1	536	29.2	641	24.3
2	League Of Legends	RTS	112	14	440	24	552	21
3	Sudden Attack	FPS	76	9.5	188	10.3	264	10
4	Talesrunner	Racing	79	9.9	136	7.4	215	8.2
5	FIFA ONLINE3	Sports	36	4.5	98	5.3	134	5.1
6	Minecraft	Arcade/Shooting	94	11.8	26	1.4	120	4.6
7	Maple Story	RPG	36	4.5	72	3.9	108	4.1
8	FIFA	Sports	24	3	80	4.4	104	3.9
9	Kart Rider	Racing	45	5.6	53	2.9	98	3.7
10	Crazy Arcade	Arcade/Shooting	14	1.8	56	3.1	70	2.7
11	Roblox	RPG	44	5.5	5	0.3	49	1.9
12	Crazy Shooting Bubble Fighter	FPS	31	3.9	18	1	49	1.9
13	Star Craft	RTS	16	2	28	1.5	44	1.7
14	Clash Royale	RTS	35	4.4	4	0.2	39	1.5
15	Heros of the Storm	RTS	12	1.5	25	1.4	37	1.4
16	Star Craft2	RTS	7	0.9	30	1.6	37	1.4
17	Counter Strike	FPS	9	1.1	16	0.9	25	0.9
18	Elsword	Arcade/Shooting	8	1	16	0.9	24	0.9
19	Team Fortress 2	FPS	17	2.1	6	0.3	23	0.9

### Internet gaming addiction and personality characteristics according to game genre

Again, the adolescents reported their top three mostly played games. Based on their most frequently played game, the participants were assigned to a game genre group. For example, if Respondent A ranked Maple Story as #1 and Overwatch as #2 most frequently played games, then A would be assigned to the RPG group which is the game genre of Maple Story. All the survey respondents who reported that they did not play online games in the past three months and were not addicted to games were assigned to the comparison group as a point of reference. The number of users by genre including non-gaming group are presented in Table 4 below.

**Table 4:** Number of users by genre.

Genre		RPG	FPS	RTS	Racing	Sports	Arcade/Shooting	Comparative Group
Specific games		Maple Story, Roblox	Overwatch, Sudden Attack, Crazy Shooting Bubble Fighter, Counter Strike, Team Fortress2	League of Legends, StarCraft1&2, Heroes of the Storm, Clash Royale	Talesrunner, Kart Rider	FIFA Online2, FIFA	Minecraft, Elsword, Crazy Arcade	Individuals who haven’t played games in the past 3 months and are not game addicted
Users	Male	91	350	396	42	104	80	413
	Female	38	126	25	120	5	79	891
	Total	129	476	421	162	109	159	1304
	(%)	(4.7)	(17.2)	(15.3)	(5.9)	(3.9)	(5.8)	(47.2)

NOTE: The comparative group consisted of 1,304 non-gaming students (789 elementary school; 515 middle school). The gaming group for the study’s analysis consisted of 1,456 students who indicated the top 6 genres in this table as their ‘game most played.’ 457 students mostly played other non-popular game genres and were excluded from our main analyses. The numbers in each game genre include all players who play the game genre regardless of their game addiction level.

#### Internet gaming addiction

A one-way analysis of variance (ANOVA) was conducted to see if there are differences among groups in their MGUS total and subfactor scores. Homogeneity of variances via Levene’s test was not met. The results of Welch’s t-test confirmed that the mean differences existed among groups. Specifically, because the assumption of equal variances was not established, post-hoc tests using the Games-Howell method were conducted to determine which groups showed statistically significant differences. First, the MGUS total scores of all game genre groups were significantly higher than that of the comparison group. Game users also had higher scores in most of the MGUS subfactor scores than the non-gamers. When comparing among genres, RTS users had significantly higher MGUS total score than Racing users. As for the MGUS subfactor scores, RTS gamers had significantly higher Tolerance scores than Racing, RPG, Arcade/Shooting gamers; FPS gamers had significantly higher Tolerance scores than Arcade/Shooting gamers. For Withdrawal, RTS gamers had significantly higher scores than Racing and RPG gamers; FPS gamers scored significantly higher than Racing gamers. For Neglect of Everyday Life, RTS gamers scored significantly higher than RPG, Racing, Arcade/Shooting gamers; FPS gamers scored significantly higher than RPG gamers. MGUS total and subfactor scores by game genre are presented in Table 5 below.

**Table 5:** MGUS total and subfactor scores by game genre.

	1		2		3		4		5		6		7		F (6, 2692–2746)	p	Post-Hoc
	M	SD	M	SD	M	SD	M	SD	M	SD	M	SD	M	SD			
MGUS Total	32.92	10.66	34.28	11.35	35.36	11.15	32.35	10.36	33.78	10.64	32.68	11.82	27.89	8.34	46.159	.000	all genre>7***
																	3>4*
Tolerance	4.71	1.64	5.15	1.93	5.31	1.95	4.63	1.58	5.11	1.85	4.61	1.79	4.00	1.34	52.459	.000	all genre>7***
																	3>1*,4***,6***
																	2>4*
Withdrawal	4.10	1.62	4.51	1.78	4.63	1.84	3.97	1.41	4.49	1.62	4.26	1.76	3.71	1.23	30.043	.000	2,3,5,6>7***
																	3>1*,4***
																	2>4***
Excessive Time Spent	5.57	2.22	5.55	2.26	5.72	2.23	5.57	2.31	5.49	2.03	5.35	2.19	4.50	1.84	31.853	.000	all genre>7***
Regulation Damage	4.99	2.30	4.95	2.13	5.20	2.16	4.80	2.18	5.00	2.11	5.04	2.35	4.09	1.67	26.658	.000	all genre>7***
Compulsive Use	5.00	2.02	5.10	1.99	5.16	1.83	4.71	1.76	4.81	1.72	4.85	1.98	4.11	1.49	33.468	.000	all genre>7***
Neglect of Everyday Life	3.79	1.13	4.27	1.45	4.39	1.57	4	1.34	4.17	1.36	3.94	1.55	3.57	1.04	32.780	.000	2,3,4,5>7***
																	3>1***,4*,6*
																	2>1***
Continued Use despite Side Effects	4.74	1.80	4.76	1.78	5.06	1.93	4.60	1.79	4.82	1.82	4.70	2.00	3.94	1.39	35.231	.000	all genre>7***

Note: Welch F is presented to correct this measure because it does not meet the equal variance assumptions.

1 = RPG, 2 = FPS, 3 = RTS, 4 = Racing, 5 = Sports, 6 = Arcade/Shooting, 7 = Comparison Group.

#### Personality characteristics

Additional ANOVA was conducted to see if any differences in individual personality characteristics exist among players of different game genre. First, Levene’s test confirmed the homogeneity of groups, and the F values indicated that statistically significant differences exist among groups. Specifically, post-hoc tests using the Bonferroni method was conducted to determine which groups showed differences. The non-gamers in the comparison group had significantly higher APQ total scores than Racing gamers. As for the APQ subfactor scores, the comparison group had significantly higher Self-Esteem scores than Arcade/Shooting group; higher Conscientiousness scores than Racing gamers; higher Empathy scores than RPG gamers; and higher Sense of Community scores than RTS and Racing gamers. Detailed statistics on APQ total and subfactor scores by game genre is presented in Table 6.

**Table 6:** Adolescent Personality Questionnaire total and subfactor scores by game genre.

	1		2		3		4		5		6		7		F(6, 2753)	p	Post-Hoc
	M	SD	M	SD	M	SD	M	SD	M	SD	M	SD	M	SD			
APQ Total	68.58	13.86	70.59	14.69	70.02	16.32	67.20	13.61	70.60	12.32	68.27	15.99	71.37	15.09	3.036	.006	7>4**
Self-Esteem	12.34	2.90	12.35	2.94	12.36	3.11	11.86	2.76	12.50	2.93	11.76	3.30	12.62	3.08	3.238	.004	7>6**
Openness	11.21	3.15	11.45	2.94	11.30	3.19	10.67	2.96	11.23	2.57	11.18	3.17	11.32	3.21	1.326	.242	
Conscientiousness	11.11	2.59	11.07	2.77	11.22	3.02	10.56	2.76	11.37	2.55	11.01	3.12	11.35	2.97	2.246	.036	7>4**
Social Leadership	9.76	3.25	10.67	3.12	10.47	3.27	9.93	2.97	10.78	2.61	10.03	3.18	10.49	3.28	2.738	.012	
Empathy	11.55	2.74	12.15	2.81	11.97	3.03	11.80	2.80	11.87	2.41	11.66	3.02	12.38	2.92	3.824	.001	7>1***
Sense of Community	12.58	2.74	12.88	2.78	12.67	3.01	12.35	2.58	12.82	2.36	12.61	2.98	13.19	2.83	4.357	.000	7>3*,4**

1 = RPG, 2 = FPS, 3 = RTS, 4 = Racing, 5 = Sports, 6 = Arcade/Shooting, 7 = Comparison Group.

## Discussion

This study was conducted with the aim of identifying how online gamers’ Internet gaming addiction characteristics and personality characteristics vary depending on the game genre they mostly play. Below, discussion of research results is presented in two parts: results related to Internet gaming addiction characteristics and those related to personality characteristics.

As for the results related to Internet gaming addiction characteristics, the total score of Internet gaming addiction was significantly higher for gamers than non-gamers, regardless of the genre. Tolerance, Excessive Time Spent, Regulation Damage, Compulsive Use, Continued Use despite Side Effects subfactor scores were also significantly higher among gamers than non-gamers in all game genres. This finding is generally supported by existing studies that also found that the use of games can cause addiction regardless of the game genre, and that preventive approach to Internet gaming addiction via promoting healthy management of game use is crucial. In particular, effective and universal preventive measures are needed for teenage youth who are prone to Internet gaming addiction [57]. A good example of this is the Internet addiction prevention program that has been in place since 2007 at the IWill Center, an Internet addiction prevention center in Seoul, Korea [58]. With governmental support, experts visit elementary and secondary schools in Seoul to conduct a six-session program that helps students gain awareness of the Internet addiction symptoms, explore alternative activities, and enhance self-control through pledges. Similar preventative efforts need to take place for Internet gaming addiction. Prevention approach model for Internet gaming has also been quite successful in Korea [59], and several government agencies have been running youth programs that have been proven effective for years [60, 61].

The results of the study also illuminated differing addiction symptom profile each game genre group is likely to show. RTS and FPS gamers showed significantly higher levels of Tolerance, Withdrawal, and Neglect of Everyday Life than other genre users. Previous studies have also reported that FPS and RTS have a higher percentage of high-risk addicts than other game genres [40] and experience higher levels of tolerance and withdrawal symptoms [39]. These results can be interpreted in several ways. First, studies have shown that rewards or achievements in games as well as social aspects of games contribute to excessive game use, which can be seen as the basic features of RTS and FPS genres [18, 35, 62]. Second, given the fact that ‘presence’ affects game immersion [63], today’s advances in computer specifications that offer gaming situations where players communicate with each other in real time with vivid sound and graphics may cause players to become more immersed in these game genres. In particular, FPS games, which are played from a first-person point of view, can cause users to identify themselves with game characters [64] or even create a transference phenomenon where users project themselves onto game characters [65]. In such cases, gaming can serve as a means to discharge their emotions and make players become more immersed in the game. Combining with new media such as virtual reality can maximize the immersion experience of FPS games. To date, however, not enough research has been done on the nature of FPS games; defining and dealing with the particular addiction problem of FPS genre seems to be an urgent task.

As for the results related to personality characteristics, Racing game users were significantly lower in Conscientiousness than non-gamers. Highly conscientious children are thought to have self-control abilities because they are sensible and goal-oriented, have a strong desire to achieve, and perform their tasks without procrastinating; thus, they are not easily addicted to the Internet [66]. Differently put, students with low conscientiousness are more likely to be attracted to gaming that can easily and quickly satisfy their needs–particularly to those game genres that can give them a sense of achievement without great effort.

Racing and RTS gamers had significantly lower sense of community than non-gamers. Given that the sense of community here refers to adolescents’ sense of connectedness with other members in their school settings, it can be low among addicted gamers. Caplan et al. [67] reported that people with high gaming addiction scores have a high sense of community online but have a low sense of community in real life. This could be because positive feedback and outcomes in the virtual world reduce the quality and quantity of community life in real life and eventually increase the sense of separation from reality-based communities [68]. This trend has emerged in participants who mostly play games of Racing and RTS genres. Games in these genres give players ranks or ratings based on individual victories, and users with many wins are recognized in the gaming world. Their network-based presence in the virtual world causes them to become more immersed in gaming, and eventually lead them to neglect real-life relationships.

Arcade/Shooting gamers appeared to have significantly lower self-esteem than non-gamers. Previous studies that examined the relationship between self-esteem and gaming reported a correlation between low self-esteem and gaming addiction [69], and suggested that problems with self-esteem may lead to gaming addiction problems [70–72]. Therefore, low self-esteem can be seen as a major predictor of Internet gaming addiction [73]. In particular, games in the Arcade/Shooting genre require simple manipulation or uncomplicated strategies, and the outcome is determined through repeated performance in a short period of time. In essence, it is relatively easy to experience a sense of achievement in Arcade/Shooting games compared to other games. In other words, adolescents who have difficulty achieving in real life may play games in order to compensate for their low self-esteem, especially through relatively easy-to-win Arcade/Shooting games [74].

RPG gamers had significantly lower Empathy scores than non-gamers. The ability to understand others have been studied as an antecedent or an outcome factor of Internet gaming addiction. In other words, individuals with low empathy tend to find it difficult to have a deep relationship with others, and are exposed to a greater risk for gaming addiction [75, 76]. As adolescents with low empathy spend more time playing games, they miss out on the opportunity to relate and understand others in the real world. This can lead to a vicious circle in which the opportunity to develop skills to empathize with others is lost again [77, 78]. On the other hand, some studies have pointed out that MMOPRG games are based on social relationships, and suggested that social relationships in the virtual world may improve players’ competence in the real world such as leadership and self-understanding [24, 79]. However, social relationships in the virtual world, especially in games, are based on factors of ability, such as how good of a game player on is rather than on one’s personal characteristics. In this context, gamers’ social skills may be developed, but the ability to empathize with others through human-to-human interaction is probably difficult to nurture. Jang et al. [23] reported that high levels of aggressiveness and greater need for reality avoidance are significant predictors of gaming addiction in RPG games. A gamer with an aggressive tendency is more likely to experience problems in real-life relationships, which can naturally lead her to avoid reality and be immersed in the game world. In such cases, game users isolated themselves and neglect their real-life relationships, which in turn can lead to a decline in their empathy levels [77].

### Future studies

In sum, differences in addiction and personality characteristics among players of different game genres call for differentiation of interventions for prevention and treatment of Internet gaming addiction. For example, addicted RTS or FPS gamers need interventions that focus more on their symptoms of Tolerance, Withdrawal, and Neglect of Everyday Life. In the meanwhile, addicted Arcade/Shooting, Racing, and RPG gamers might need to focus on enhancing their relatively weak personality traits, such as conscientiousness, sense of community, self-esteem and empathy, rather than directly deal with addiction-related problem behaviors. While it is difficult to explain the complex phenomenon of Internet gaming addictions simply with the six APQ personality traits, the discriminatory characteristics that were associated with each game genre in this study may be considered in connection with other widely studied individual traits such as sensation seeking or impulsiveness to gain a better understanding of the phenomenon [35, 80].

### Limitations

There are several limitations in this study. First, it must be noted that this study was done on adolescents in Korea. Generalization of the results must be heeded; they must be interpreted with caution by taking into consideration of the cultural and social characteristics of students going through adolescence in Korea. Comparative international studies should follow to investigate the impact of culture and society on the differences among different game genres. Second, the question of whether the current categorization of game genres can encompass all future games arises. Although existing popular online games can be categorized into a genre relatively easily, games that have a mix of features from multiple genres are increasing. Thus, Internet games developed in the future are likely to further obscure these genre-specific classifications. Therefore, it is necessary to develop a more sophisticated system for classifying games. The systematic and scientific classification of games will greatly help develop more effective, individualized treatment or prevention strategies. Third, the popularity of particular games in Korea at the time of study may have influenced the results. There may have been relatively fewer RPG users than usual at the time of the data collection due to an emergence of certain RTS and FPS games. Genre-specific research will better clarify the characteristics of each genre. Fourth, the decision to group the participants into genres based on their #1 reported game does not account for the possibility of a user who spend similar amount of time playing their #1 and #2 games of two different genres. Although subjective reporting of the game most played may prove to be meaningful, future studies could take measures to control for this kind of confound.
